# Vascular mechanism of axonal degeneration in peripheral nerves in hemiplegic sides after cerebral hemorrhage: An experimental study

**DOI:** 10.1186/1749-7221-3-13

**Published:** 2008-04-28

**Authors:** Cemal Gundogdu, Memet Dumlu Aydin, Dilcan Kotan, Nazan Aydin, Ednan Bayram, Hızır Ulvi, Recep Aygul

**Affiliations:** 1 Department of Pathology, Medical Faculty, Ataturk University, Erzurum, Turkey; 2 Department of Neurosurgery, Medical Faculty, Ataturk University, Erzurum, Turkey; 3 Department of Neurology, Medical Faculty, Ataturk University, Erzurum, Turkey; 4 Department of Psychiatry, Medical Faculty, Ataturk University, Erzurum, Turkey; 5 Cardiology Clinic of Erzurum State Hospital, Erzurum, Turkey

## Abstract

**Background:**

Though retrograde neuronal death and vascular insufficiency have been well established in plegics following intracerebral hemorrhage, the effects of plegia on arterial nervorums of peripheral nerves have not been reported. In this study, the histopathological effects of the intracerebral hemorrhage on the dorsal root ganglions and sciatic nerves via affecting the arterial nervorums were investigated.

**Methods:**

This study was conducted on 13 male hybrid rabbits. Three animals were taken as control group and did not undergo surgery. The remaining 10 subjects were anesthetized and were injected with 0.50 ml of autologous blood into their right sensory-motor region. All rabbits were followed-up for two months and then sacrificed. Endothelial cell numbers and volume values were estimated a three dimensionally created standardized arterial nervorums model of lumbar 3. Neuron numbers of dorsal root ganglions, and axon numbers in the lumbar 3 nerve root and volume values of arterial nervorums were examined histopathologically. The results were analyzed by using a Mann-Whitney-U test.

**Results:**

Left hemiplegia developed in 8 animals. On the hemiplegic side, degenerative vascular changes and volume reduction in the arterial nervorums of the sciatic nerves, neuronal injury in the dorsal root ganglions, and axonal injury in the lumbar 3 were detected. Statistical analyses showed a significant correlation between the normal or nonplegic sides and plegic sides in terms of the neurodegeneration in the dorsal root ganglions (p < 0.005), axonal degeneration in the lumbar 3 nerve roots (p < 0.005), endothelial cell degeneration in the arterial nervorums (p < 0.001), and volume reduction in the arterial nervorums (p < 0.001).

**Conclusion:**

Intracerebral hemorrhage resulted in neurodegeneration in the dorsal root ganglion and axonolysis in the sciatic nerves, endothelial injury, and volume reduction of the arterial nervorums in the sciatic nerves. The interruption of the neural network connection in the walls of the arterial nervorums in the sciatic nerves may be responsible for circulation disorders of the arterial nervorums, and arterial nervorums degeneration could result in sciatic nerves injury.

## Introduction

The peripheral nerves are supplied by arterial nervorums (ANs) and innervated by neural networks localized in the perivascular spaces. ANs are connected to each other with many anastomoses [8,9]. Autoregulation of nerve blood flow of peripheral nerves (PNs) is impaired and results in hypotension in the plegic side [10-12]. Decreased blood flow in ANs may result in degeneration of nerve fibers and loose of the myelin sheath [13]. Spinal cord injury results in impaired vascular control and circulation disorders at the extremities [2]. Nerve, muscle, and vascular atrophy are even possible after spinal cord injury [3,4]. Disordered central and spinal autonomic reflexes seem to play an important role in PN injury and polyneuropathy [5-7]. The diameters and blood flow velocity of the femoral arteries are significantly reduced in the paralytic site [1]. The femoral arteries are innervated by the L_1–6 _segments of the spinal nerves in animals [14]. Intracerebral hemorrhage (ICH) causes descending neurodegeneration from the cortex to the dorsal root ganglion (DRG) [15]. Then, ICH causes destruction of the reflex arches of the ANs due to neurodegeneration in the DRG of L_1–6_. Although PNs injury has been reported as a cause of power loss at the involved muscles, injury to the feeding vessels of the PNs has not been investigated in hemiplegic subjects after cerebral hemorrhage. In this study, we aimed to prove that the hemiplegia due to ICH results in histopathological changes in the ANs of the PNs.

## Materials and methods

In the present study were included 13 male hybrid rabbits. Animals were 2 years old and weighed approximately 4 kg each. Animal experimentation was carried out according to the guidelines set by the ethical committee of our university. All animals were anaesthetized by subcutaneous injection of a mixture of ketamine hydrochloride (25 mg/kg), lidocaine hydrochloride (15 mg/kg), and acepromasine (1 mg/kg). After preparation of the operative site, a left parietal burr-hole of 3 mm diameter was created, and 0.25 cc venous blood from the same animal was injected into the right sensory-motor cortex. After the operation, the fascia and skin were sutured by 4.0 absorbable suture material. The rabbits were followed in their personal cages and given antibiotic (cefotaxime 125 mg/BID) and analgesic (methamisozol sodium 10 mg/kg) therapy for six days postoperatively. One month later, all animals were sacrificed, and their lumbar 3 (L_3_) nerve roots were removed bilaterally. For light microscopic analysis, these specimens were preserved in 10% formalin solution. These specimens were embedded in paraffin blocks, and sections were stained with hematoxyline and eosin. ANs and L_3 _roots were evaluated. The numbers of the normal and degenerated axons were determined, and the ANs were evaluated in all roots. Axonal degeneration criteria were defined as axonolysis or axonal loss, periaxonal halo formation, and Schwann cell necrosis. AN degeneration criteria were defined as endothelial cell shrinkage, angulaton, cell necrosis or loss, muscular thinning, and intimal edema. The Cavalieri volume estimation method was used to obtain the total number of axons in each nerve root (NR). The total number of axons was estimated by multiplication of the volume (sample item area) and the numerical density of neurons in each L_3 _NR. The statistical comparison was performed between the paraplegic and contralateral side roots at the L_3 _level.

In histopathological examination, cytoplasmic condensation, cellular shrinking, cellular angulations secondary to cytoplasmic regression, endothelial cell loss, was accepted as the both endothelial and neuronal degeneration criteria. Also axonolysis, axonal loss, periaxonal halo formation and myelin loss were accepted as the axonal degeneration criteria. All of the degenerative findings were more prominent on the plegic side than on the non-plegic side.

**Figure 1:**
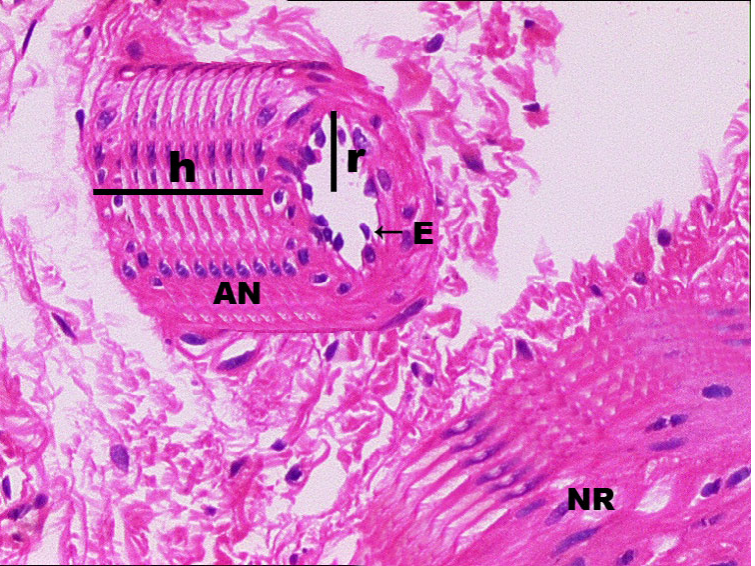
A nerve root and its supplying artery of a normal rabbit are represented, which were reconstructed three dimensionally by using consecutive sections of the same artery specimen. The reconstructed artery is accepted as a cylinder, and its surface area is calculated as: S = 2∏rh; the endothelial cell density was calculated in a part of ANs. Volumetric changes of the ANs were calculated as the volume of the cylinder-shaped artery by the following formula: V = ∏r^2^h. Vascular luminal changes of the arteries were calculated by using volume changes of the ANs instead of changes in vessel diameter.

Endothelial cell were arranged in the surface of the inner cavity of cylindiric. Endothelial cells arranged plane originally is a rectangle which forming inner surface of the cylindrical inner cavity of ANs. The borders long of the reference cylinder are given by **2 ∏r **and **h**. Thus, the surface area of the reference plane is calculated by the following equation: **S **= **2 ∏r **× **h**. In the same way, the number of endothelial cells was estimated in each reference plane and accepted as the endothelial cells density (Figure 1). To calculate the volumetric changes of the ANs due to vasospasm or vasodilatating factors, a three-dimensional cylindrical AN model was created by the reconstruction of seven consecutive hystological sections of each ANs (Figure 1). In the AN model, the luminal radius is represented by 'r', and the height is represented by 'h'. Geometrical volume calculation methods were used in the reconstructed cylindirical ANs sample. The standardized ANs volume was calculated with the following formula:

V = ∏r^2^h

Statistical analysis was performed using a nonparametric Mann Whitney-U Test.

## Results

**Figure 2:**
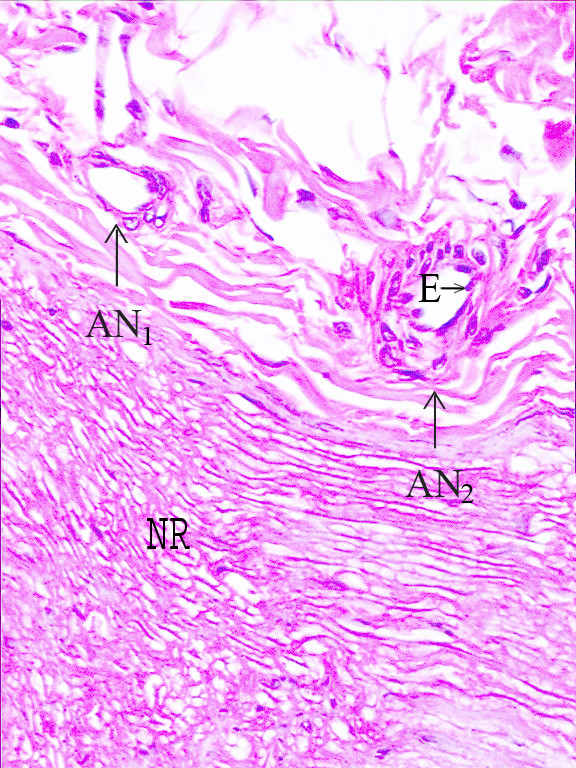
Normal appearance of a nerve root and epineural arteries at the level of L_3 _(H&E, ×100, LM). (AN_1,2_: Arterial nervorum, NR: Nerve Root, E: Endothel) (H&E, ×100, LM).

**Figure 3:**
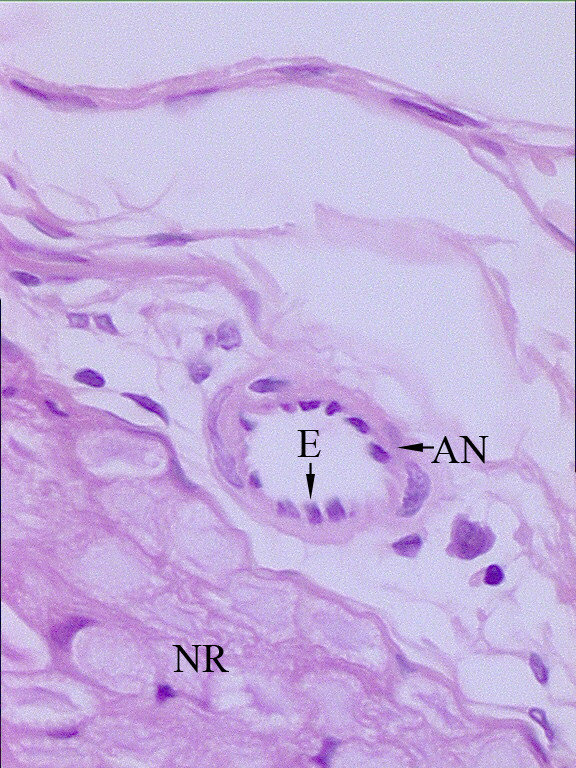
Appearance of a nerve root (NR), arterial nervorum (AN), and endothelial cells of the ANs (E) at the L_3 _level on the non-plegic side. Minimallly endothelial swelling, cellular loss, and axonal injury are observed (H&E, ×200, LM).

**Figure 4:**
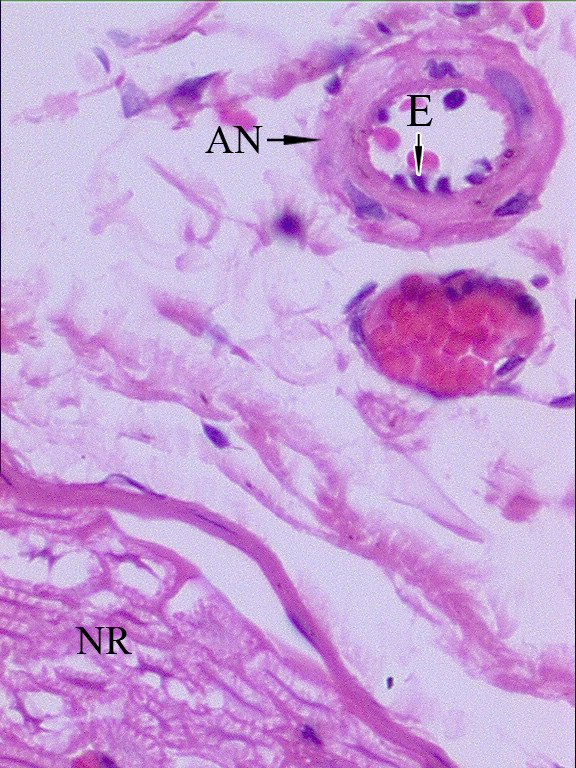
Appearance of a nerve root (NR), arterial nervorum (AN), and endothelial cells of the ANs (E) at the L_3 _level on the non-plegic side. Endothelial shrinkage, angulation, and cellular loss are seen in ANs of the nerve root at the level of L_6 _on the plegic side (H&E, ×100, LM). Degenerated axons, myelin sheath derangements, and axonal loss are seen on the plegic side (H&E, ×200, LM).

Left hemiplegia developed in eight animals. The histological appearance of the normal rabbit NR and ANs is shown in Figure 2. Figure 3 shows a histopathological representation of an NR and AN on the nonplegic side, and Figure 4 shows a NR and AN on the plegic side.

The total number of normal axons of the L_3 _anterior root was estimated as 20,000 ± 1500 in normal animals (N = 3). The number of normal axons of L_3 _was 19,700 ± 1000 on the non-hemiplegic side and 13,000 ± 700 on the plegic side. Degenerated neuron numbers were estimated as 30 ± 5 in normal subjects, 200 ± 50 on the nonplegic side, and 7000 ± 500 on the plegic side. The difference in axonal degeneration between the normal and nonplegic sides was not statistically significant (p < 0.05), but the difference between the nonplegic and plegic sides was significant (p < 0.005). The difference between the plegic and normal sides was also significant (p < 0.001).

The endothelial cell density of the ANs was about 280 ± 20 cells/item area in normal animals. The endothelial cell density of the ANs was 260 ± 15 cells/item area on the nonplegic side of experimental animals and 150 ± 30 cells/item area on the plegic side. The difference between the normal and nonplegic sides was not significant (p < 0.5), but the difference between the plegic and nonplegic sides was significant (p < 0.005). The difference between the plegic and normal sides was also statistically significant (p < 0.0001). The volume of an imaginary AN was found to be 1000 item volume in normal animals, 900 item volume on the nonplegic side and 600 item volume on the plegic side. The difference in volume reduction of the AN was significant between the hemiplegic sides and the normal or non-plegic sides (p < 0.001).

Table 1 shows the average number of normal and degenerated axons, neurons of dorsal root ganglions (DRGs), endothelial cell numbers, and volumetric changes of the AN sample in each groups. Plegia caused endothelial cell necrosis, neuronal and axonal degeneration in the DRG and sciatic nerves (SNs), and volume reduction in the AN on the plegic sides.

**Table 1:** The total number of normal and degenerated axons of L_3 _roots, the number of neurons in the DRGs, the volume values, and the number of endothelial cells of AN samples are given.

	Normal animals	Non-plegic side	Plegic side
**Number of normal neurons of a DRG**	20. 000 ± 100	19.700 ± 100	13. 000 ± 7.300
**Degenerated neuron numbers of a DRG**	30 ± 5	200 ± 50	7500 ± 500
**Normal axon numbers of an L**_3_	6. 000 ± 300	4.700 ± 200	3. 150 ± 150
**Degenerated axon numbers of an L**_3_	20 ± 5	1200 ± 50	2500 ± 200
**Number of endothelial cells of a normal AN (Cell/item area)**	280 ± 20	260 ± 15	150 ± 30
**Number of degenerated endothelial cells of ANs (Cell/item area)**	10 ± 3	20 ± 5	120 ± 10
**Volume values of a standart part of an AN (item volume).**	1000	900	600

The differences in the number of degenerated axons between the normal and non plegic groups were not statistically significant (p < 0.05). In contrast, the differences between the non-plegic and plegic groups were significant (p < 0.005), but the differences between the normal and plegic groups were most significant (p < 0.0001). As for the endothelial cells of ANs, the difference between the normal and nonplegic side was not significant (p < 0.5), and the difference between the plegic and nonplegic side was significant (p < 0.005). The differences between the plegic side and normal groups, however, were most significant (p < 0.0001).

## Discussion

The peripheral nerves are supplied by ANs and innervated by neural networks longitudinally localised in the endoneurium, perineurium, and epineurium. ANs are connected to each other and form many anastomoses in the subepineural spaces [8,9]. Epineurial vessels contain smooth muscle, have large diameters, and are innervated by somatosensitive and autonomic plexuses of unmyelinated nerve endings [6,18,20,24,25]. Endoneurial vessels, however, have no smooth muscle and neural innervation, and the endoneurial blood flow is under the influence of vasoactive substances [6,16-23]. The density of the nerve axons decreases gradually from the epineurium to the endoneurium [18,20]. A degenerated perivascular plexus may result in disordered regulation of the PNs blood flow and result in PNs damage [31].

The diameters and blood flow velocities of the common femoral arteries decrease significantly secondary to inactivity of the paralytic state, and this process is largely completed within weeks [1]. Spinal cord injury results in impaired vascular control, circulation disorders, and muscle atrophy [2,3]. Transient spinal cord ischemia causes degenerative changes in the motor and mixed PNs, with partial or total plegia [4]. Disordered centrospinal sympathetic veno-arteriolar or myogenic reflexes play an important role in the development of PNs injuries [5].

Impaired innervation of blood vessels of PNs in patients with diabetes mellitus has been associated with the development of detrimental peripheral arterial disease [6,7]. Unfortunately, autoregulation of ANs can be corrupted and nerve blood flow can be reduced during hypotension in plegic conditions [10-12]. Decreased blood flow in PNs may result in degeneration of nerve fibers and loss of the myelin sheath. Also, neuropathic features are triggered in relation to the severity of ischemia in patients with peripheral arterial disease. Decreased innervation of ANs could lead to a disturbed oxygenation of the PNs and development of neuropathy [13].

The existence of circulatory disturbances [26] and large myelinated fiber loss in the nerve roots of plegics is well established [27]. In such cases, neuronal death begins within the first day and mostly progresses within the first 2 months, and cell death is limited up to 6 months [28]. Severely damaged neurons and axons in PNs have also been observed in complicated cerebro-spinal traumas [29]. Neuronal degeneration of the PNs has been reported in autopsy studies of patients with perinatal hemorrhagic telencephalic necrosis [30]. Hemorrhagic lesions of the sensory motor cortex commonly cause power loss at the involved muscles, but feeding vessels of PNs has not been reported in plegic subjects.

It has been reported that brain or spinal cord injuries cause neuronal degeneration in spine ganglia and axonal degeneration in PNs by the mechanism of proximal axotomy [29]. Seven days after cerebral or spinal cord injuries, 24% of the dorsal root ganglion neurons were lost, and 54% were lost 28 days after axotomy [32]. The physical proximity of the lesion to the cell body is a critical factor for the development of PNs injury [33]. The microscopic and ultra-structural changes indicate that there are typical morphological changes similar to those of apoptosis, including condensed basophilic nuclei, formation of nuclear caps, cell shrinkage, and apoptotic body formation following sciatic nerve axotomy [34]. In degenerative disease of the brain and spinal cord, myelin loss with segmental demyelination and axonal degeneration has been observed in sensory and motor fibers of PNs [35].

In this study, we aimed to prove whether hemiplegia due to ICH may result in histopathological changes in ANs. It is not known whether the disordered blood flow of ANs causes neuronal degeneration in PNs after ICH. For this reason, we investigated the histomorphological changes of axons of the spinal nerve roots (NRs) on each side of normal rabbits. In our experiment, the centrally axotomised PNs model was created through intracranial hemorrhage as described by Taiushev [29]. It has been shown previously that circulation disorders of ANs may result in PNs degeneration [13] and that ICH causing hemiplegia results in descendent degeneration from cortex to DRG [15]. Because the femoral arteries are innervated by the L_1–6 _segments of the SNs [14], the hemiplegic condition may affect the neural innervation of the femoral arteries.

To estimate the number of normal or degenerated neurons in each DRGs and PNs, we used stereological methods described in previous studies [15,36-38]. In our study, intracerebral hemorrhage may have caused the destructions of reflex arches of ANs via its degenerative effects on the DRGs of L_1–6_. Descending neurodegeneration of sensitive reflex pathways of ANs in SNs may be destroyed, and circulation disorders of ANs in SNs may be inevitable. Eventually, decreased blood flow in the ANs may result in degenerative changes in nerve fibers of SNs.

According to our experiments, ICH resulted in neurodegeneration and axonolysis in PNs, vascular injury, and volume reduction of the ANs. The interruption of the neural networks in the walls of the ANs may be responsible for circulation disorders of the ANs. Consequently, ANs degeneration could result in PNs injury.

In summary; by creating a centrally axotomised model through a hemorrhagic sensory-motor cortex lesion, endothelial cell injury, neuronal and axonal degeneration may occur in the PNs on the plegic sides. In the aetiology of PNs degeneration in plegic sides after intracerebral hemorrhage ANs injuries should be considered as an important factor.

## Authors' contributions

MDA the pathological processes. MDA, NA, DK performed experiment procedure and surgery. CG evaluated histopathology. HU and RA conducted clinical evalution and interpreted results. EB explain peripheral vascular function All authors read and approved the final manuscript.
